# Genomic insights into selective signals and local adaptation of a litchi subspecies

**DOI:** 10.1186/s43897-025-00205-8

**Published:** 2026-03-09

**Authors:** Pengfei Wang, Xueren Cao, Hui Zhang, Huanling Li, Huiyun Zhang, Songgang Li, Jiwang Hong, Jian Zheng, Xinping Luo, Chengjie Chen, Lei Zhang, Jiabao Wang

**Affiliations:** 1 Tropical Crop Genetic Resources Institute, Key Laboratory of Crop Gene Resources and Germplasm Enhancement in Southern China, Ministry of Agriculture and Rual Affairs, Key Laboratory of Tropical Crops Germplasm Resources Genetic Improvement and Innovation of Hainan Province, Chinese Academy of Tropical Agricultural Sciences, Haikou, 571101 China; 2 Environment and Plant Protection Institute, Chinese Academy of Tropical Agricultural Sciences, Haikou, 571101 China; 3 Tropical and Subtropical Cash Crops Research Institute, Yunnan Academy of Agricultural Sciences, Baoshan, 678000 China

Litchi (*Litchi chinensis* Soon.) is an important economic crop in tropical and subtropical regions, cultivated extensively across 35 countries worldwide. This homogeneous production structure presents several challenges for the litchi industry, including limited risk resistance, a lack of market diversification, and difficulties in extending the production period (Chen et al. 2024). In Yunnan, two variants of wild litchi, *Litchi* *chinensis* var. *spontaneous* and *Litchi chinensis* var. *fulvosus* have been identified, of which *L. chinensis* var. *fulvosus* exhibits ideal agronomic traits, including early flowering, early fruit maturity, multi-season flowering and high fruit setting rate (Zhang et al. 2020). While these traits make *L. chinensis* var. *fulvosus* a valuable genetic resource for breeding ideal litchi cultivars, its population dynamics and genetic architecture remain underexplored.

In this study, we collected 192 *L. chinensis* var. *fulvosus* individuals from a wide geographical range in Yunnan, along with 178 other litchi genetic resources from Guangdong, Guangxi and Hainan. We analyzed the distinct genetic structure of *L. chinensis* var. *fulvosus*, identified genomic selective signals associated with its superior traits, examined the genetic basis of the local adaptability, and predicted the genetic vulnerability of the *L. chinensis* var. *fulvosus* population under future climate change conditions.

A total of 192 *L. chinensis* var. *fulvosus* individuals distributed along the Yuan River in Yunnan and 178 litchi resources were collected from Guangxi, Guangdong and Hainan in China (Fig. 1A, S1; Table S1). A total of 645,576 SNPs were identified with the Litchi40K v1.0, while 28.7% were located in genic regions, 42.8% in regulatory regions, and 28.5% in intergenic regions (Zhang et al. 2025) (Fig. S2; Table S2).

**Fig. 1:**
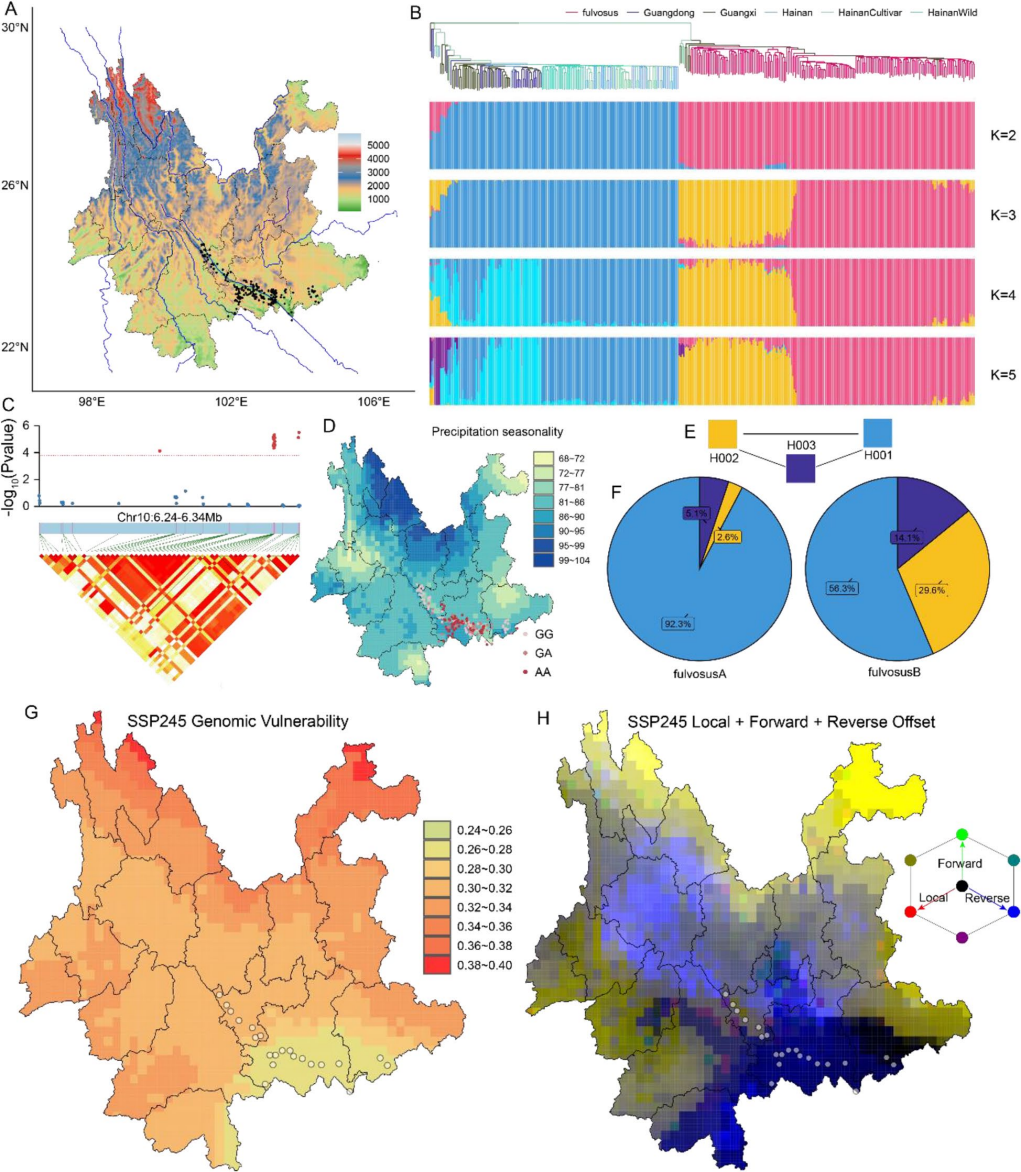
Genetic architecture and local adaptation of *L*. *chinensis* var. *fulvosus*. **A**. Sampling sites of *L. chinensis* var. *fulvosus* in Yunnan. **B**. The phylogenetic analysis and ancestral component analysis from K values 2 to 5 of the whole population. **C**. Local manhattan plot of candidate adaptive SNP nearby LITCHI022461 associated with BIO15. **D**. Allele distribution of candidate adaptive SNP associated with BIO15. **E**. Haplotype evolution path of LITCHI022461. **F**. Distribution of LITCHI022461 haplotype in two subgroups. **G**. The genetic offset under the SSP245 climate scenarios in 2081–2100. **H**. The RGB map of local (red), forward (green), and reverse (blue) offset under the SSP245 climate scenarios in 2081–2100

The phylogenetic analysis revealed two distinct clades, namely the *fulvosus* resources and the litchi resources. As the K value increased, genetic differentiation initially appeared within the *fulvosus* resources, followed by differentiation among the resources from Guangdong. The cultivars from Hainan consistently clustered together. In contrast, some individuals from Guangxi partially clustered with those from Guangdong, while others clustered with individuals from Hainan (Fig. 1B). This finding corroborated by principal component analysis (Fig. S3). Finally, the 339 individuals were divided into four subgroups, Guangdong, Hainan, fulvosusA and fulvosusB, while 31 mixed individuals were excluded (Table S3).

Among the four subgroups, the lowest nucleotide diversity was found in the fulvosusB subgroup (Fig. S4A). The Fixation index (*Fst*) value between the two subgroups of *L*. *chinensis* var. *fulvosus* and the other two litchi subgroups were all above 0.74, indicating significant differentiation between the *L*. *chinensis* var. *fulvosus* resources and other litchi resources (Fig. S4B).

Approximately 93% of deleterious variants were present in homozygous form. The Guangdong subgroup exhibited the highest number of homozygous mutations, followed by Hainan, fulvosusA, and fulvosusB (Fig. S5). The U-shaped distribution of SFS distributions in fulvosusA and fulvosusB indicates population contraction or background selection pathway, and the left-skewed distribution in Guangdong and Hainan subpopulations indicates population expansion or gene inflow pathway (Fig. S5).

A total of 112 regions and 333 genes were identified through *Fst* and XP-CLR analyses between the two subgroups of *L. chinensis* var. *fulvosus* and the two litchi subgroups (Fig. S6, S7; Table S4, S5). Several key functional genes were associated with these selection signals. Genes such as *TOC1*, *LHY*, and *GA20OX2* influence flowering time, while *NAC2* and *RCD1* are involved in abiotic stress responses, and *MYB3* and *ARF8* play roles in growth and development. Fifteen variants were identified in the *NAC2* gene region, categorizing the population into nine haplotypes (Table S6). Notably, the ancient haplotype, H001, is exclusive to the fulvosusA and fulvosusB subgroups, while the derived haplotypes H005, H003, and hybrid haplotypes H002 and H004 were dominant in the Guangdong and Hainan subgroups (Fig. S8).

A total of 1803 and 1504 selective genes were detected in the fulvosusA and fulvosusB subgroups using SweepFinder2, enriched in pathways related to the metabolism of organic hydroxy compound, glucosidase activity, and cGMP biosynthetic processes (Fig. S9).

Significant isolation by environment (IBE) and isolation by distance (IBD) were identified within *fulvosus* resources, indicating a spatial structure in the distribution of *fulvosus* resources (Fig. S10). Principal component analysis (PCA) of 19 environmental variables, including 10 temperature-related and 9 precipitation-related factors, revealed distinct ecological niches for the fulvosusA and fulvosusB subgroups (Fig. S11A). The observed similarity index (*D* = 0.45, *P* = 0.03) simulated by Environmental niche modeling further confirmed these differences (Fig. S11B).

Two complementary genotype-environment association approaches were employed to detect the environment-associated genetic variants. A total of 14,937 SNPs were identified to be associated with one or more environmental variables, which were distributed widely throughout the genome with the latent factor mixed model (Frichot and François 2015) (Table S7). 7,660 SNPs were found to display extreme loadings (standard deviation > 3) along one or more RDA axes with redundancy analysis (Capblancq and Forester 2021) (Fig. S12; Table S8). Finally, 3,079 SNPs were identified as core adaptive variants, representing the intersections between LFMM and RDA results, while a total of 815 adaptive genes were annotated and enriched in processes such as proteolysis involved in protein catabolic and ubiquitin − dependent protein catabolic processes (Fig. S13; Table S9, S10).

A total of 196 core adaptive variants were identified with temperature seasonality (BIO4). A candidate adaptive SNP was located at the anterior end of chromosome 1, *LITCHI014753* annotated as a luminal-binding protein with a temperature-responsive HSP70 domain was candidate gene with geographical distribution analysis and haplotype analysis (Vierling 1991) (Fig. S14; Table S11). A total of 70 core adaptive variants were identified with precipitation seasonality (BIO15). A candidate adaptive SNP was located at the anterior end of chromosome 10, and *LITCHI022461* annotated as a lateral organ boundaries protein was candidate gene (Coudert et al. 2015) (Fig. 1C, S15). Geographic distribution analysis revealed that the wild-type GG genotype was mainly distributed in areas with lower precipitation seasonality (Fig. 1D). Eight variants were identified in the *LITCHI022461* gene region, categorizing the population into three haplotypes (Fig. 1E; Table S12). Notably, the ancient haplotype H002 and the heterozygous haplotype H003 were dominant in the fulvosusB subgroup, while the derived haplotypes H001 was dominant in the fulvosusA subgroup (Fig. 1F).

Three complementary approaches were employed to evaluate the genetic vulnerability of *L. chinensis* var. *fulvosus* to future environmental changes (Sang et al. 2022). The *L. chinensis* var. *fulvosus* population is expected to exhibit higher the risk of non-adaptedness (RONA) values in regions experiencing more severe environmental changes (Fig. S16; Table S13). A gradient forest model was employed to predict genetic offset under future climate scenarios (Fig. S17). Across the spatial distribution of the *L. chinensis* var. *fulvosus* population, the entire population exhibited high genetic offset values (Table S14). Notably, the *L. chinensis* var. *fulvosus* population in the upper reaches of the Yuan River displayed significantly higher genetic offset values compared to those in the lower reaches (Fig. 1G, S18). In addition to assessing RONA and genetic offset, we also evaluated local, forward, and reverse genetic drift. The local and forward genetic drift values were notably higher for the *L. chinensis* var. *fulvosus* population in the upper reaches of the Yuan River (Fig. 1H, S19; Table S15).

In summary, our research established the taxonomic status of *L. chinensis* var. *fulvosus* an independent subspecies of litchi. The selection signal genes associated with ideal agronomic traits were identified in *L. chinensis* var. *fulvosus*, which can serve as valuable targets for litchi breeding programs. Additionally, we analyzed the adaptive genetic basis of the *L. chinensis* var. *fulvosus* in its current environment, offering critical insights into its ecological resilience. These findings provide an important theoretical foundation for future efforts in the conservation and potential relocation of *L. chinensis* var. *fulvosus*.

## Supplementary Information

Supplementary Material 1.

Supplementary Material 2.

## Data Availability

All raw reads generated for the individuals in the study have been deposited in the National Genomics Data Center under BioProject PRJCA026922.
