# Supplementary material for: Genomic insights into selective signals and local adaptation of a litchi subspecies
Source: Mol Hortic. 2026 Mar 9;6:23. doi: 10.1186/s43897-025-00205-8 (PMC12969909; doi:10.1186/s43897-025-00205-8)
Supplement: Supplementary file 1 — Supplementary Material 1. [file 43897_2025_205_MOESM1_ESM.docx]

**
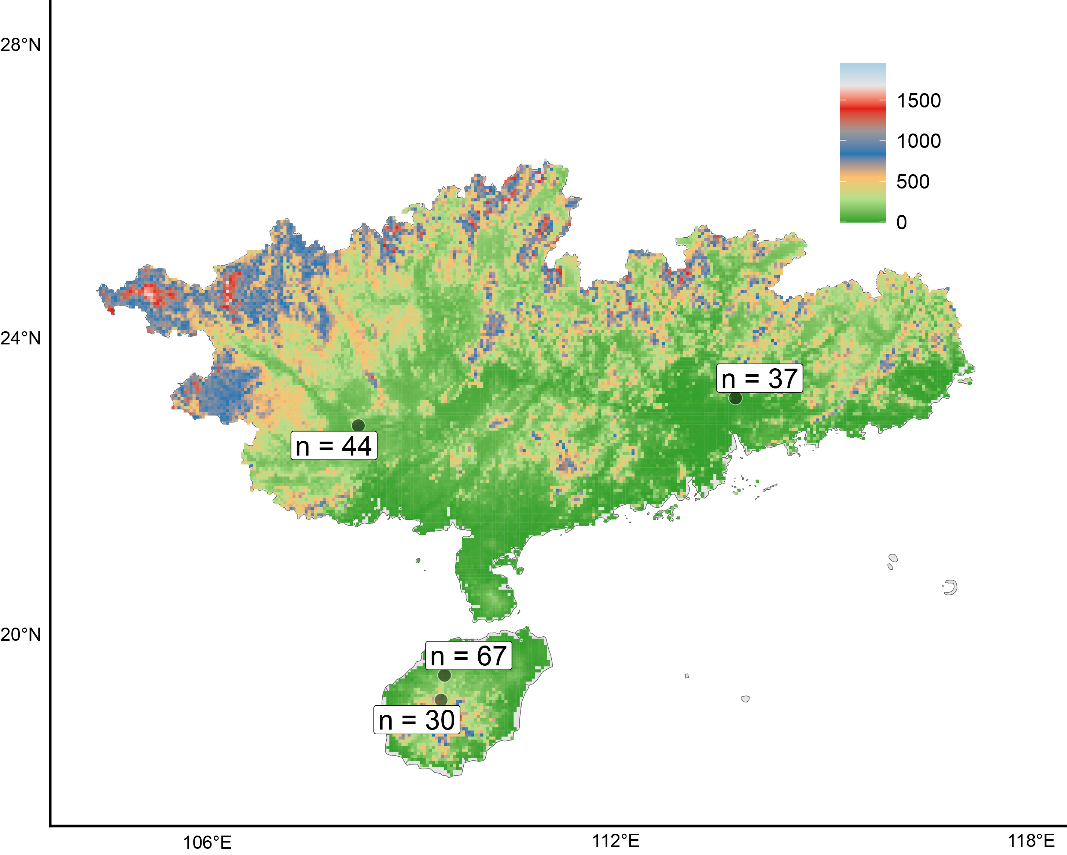
**

**Supplementary Fig. 1 Sampling sites of litchi in Guangxi, Guangdong and Hainan**

**
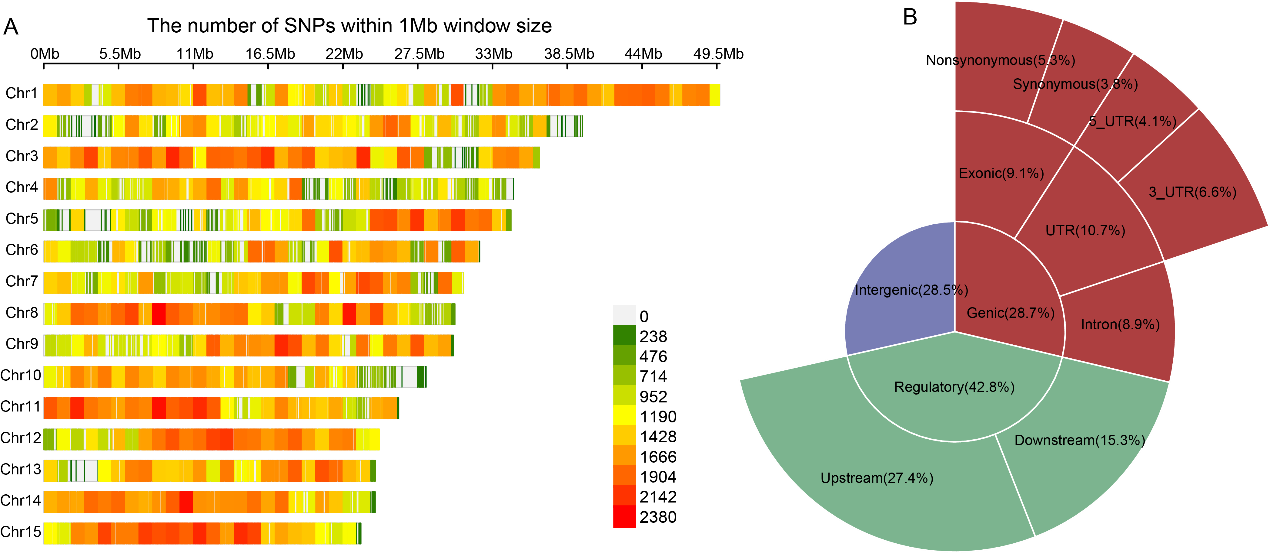
**

**Supplementary Fig. 2 Genotyping of the whole population**

**A.** The genome-wide SNP identified in the whole population. **B**. The genomic position of SNP identified in the whole population.

**
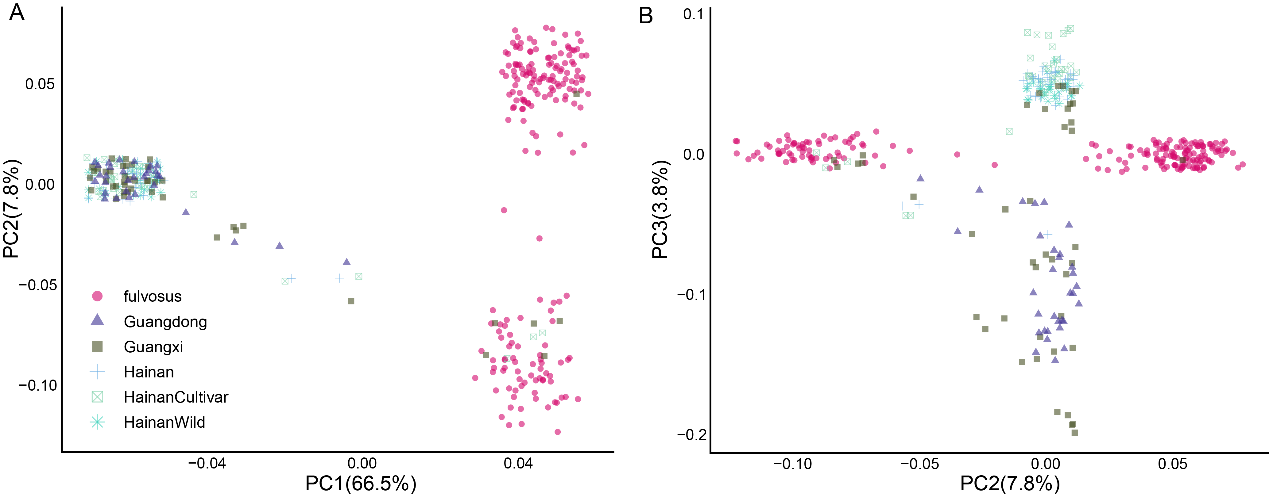
**

**Supplementary Fig. 3 Principal component analysis of the whole population**

**A**. PCA plot of the whole population with the first two PCs. **B**. PCA plot of the whole population with the second and the third PCs.

**
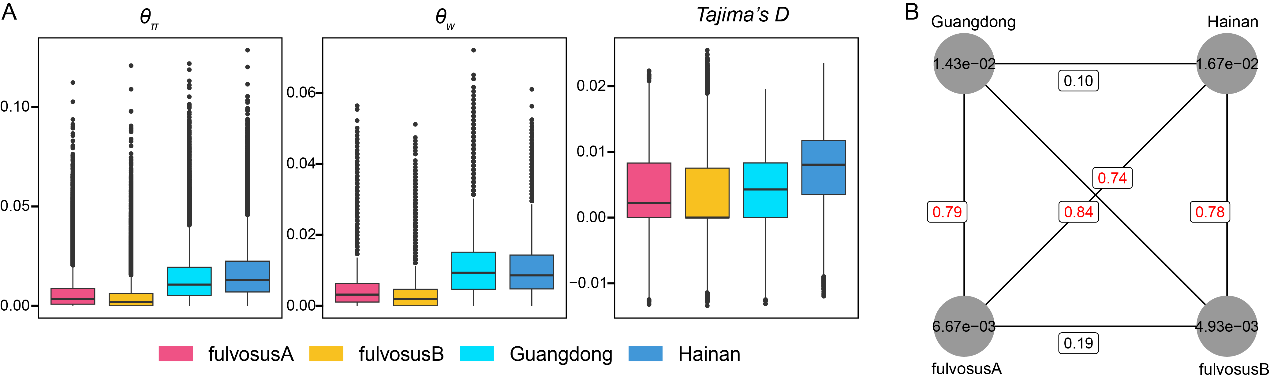
**

**Supplementary Fig. 4 Genetic diversity of the whole population**

**A**. Genetic diversity and fixation index of the four subgroups. **B**. The pairwise nucleotide diversity *θ_π_*, Watterson's estimator *θ*_W_ and Tajima's *D* of the four subgroups.

**
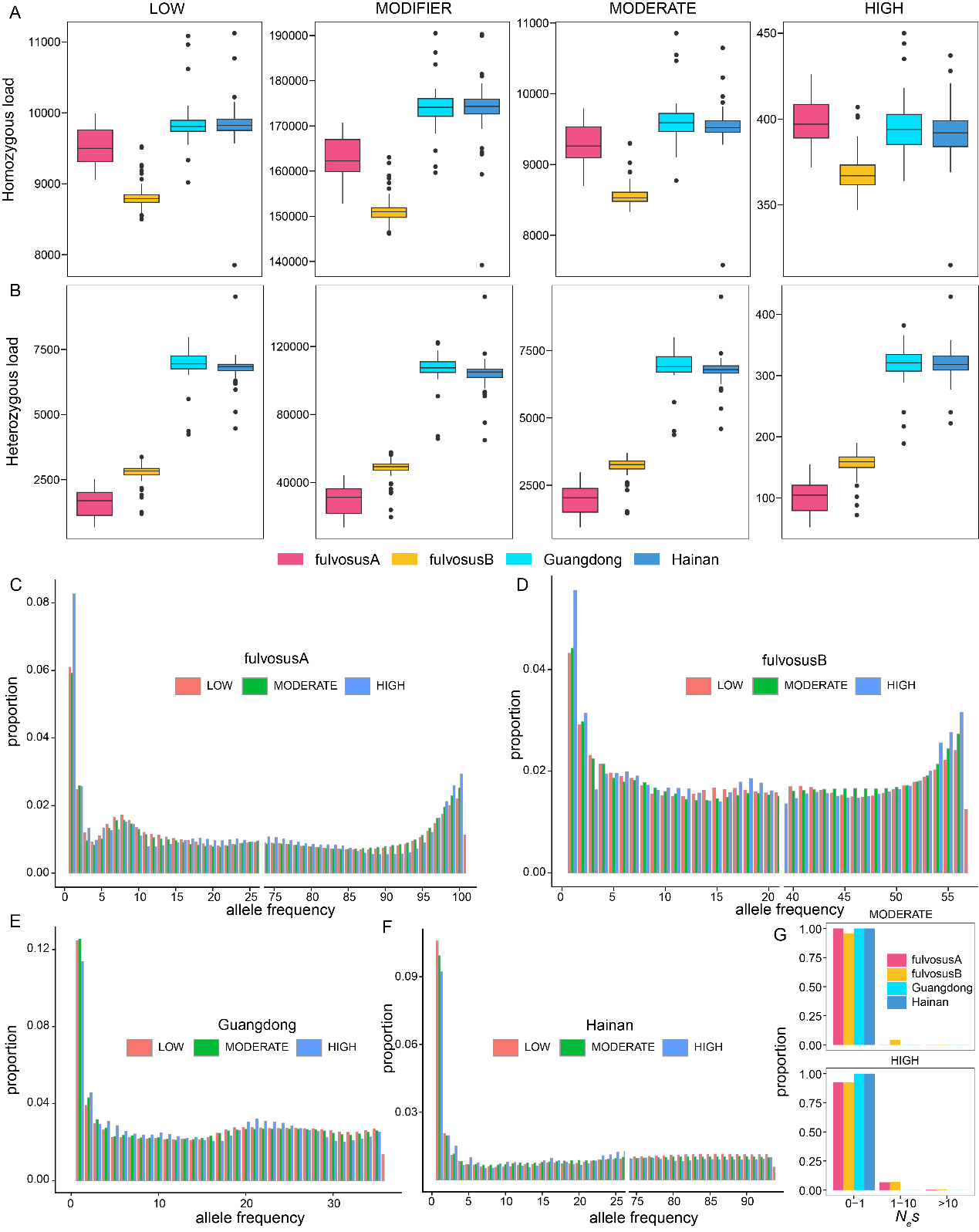
**

**Supplementary Fig. 5 Mutational load in four subgroups**

**A**. The homozygous load of genetic variants with low, modifier, moderate and high effect in four subgroups. **B**. The heterozygous load of genetic variants with low, modifier, moderate and high effect in four subgroups. **C**. The site frequency spectrum of synonymous, moderately and highly deleterious mutations in fulvosusA. **D**. The site frequency spectrum of synonymous, moderately and highly deleterious mutations in fulvosusB. **E**. The site frequency spectrum of synonymous, moderately and highly deleterious mutations in Guangdong. **F**. The site frequency spectrum of synonymous, moderately and highly deleterious mutations in Hainan. **G**. The fitness effect for moderately and highly deleterious mutations in four subgroups.

**
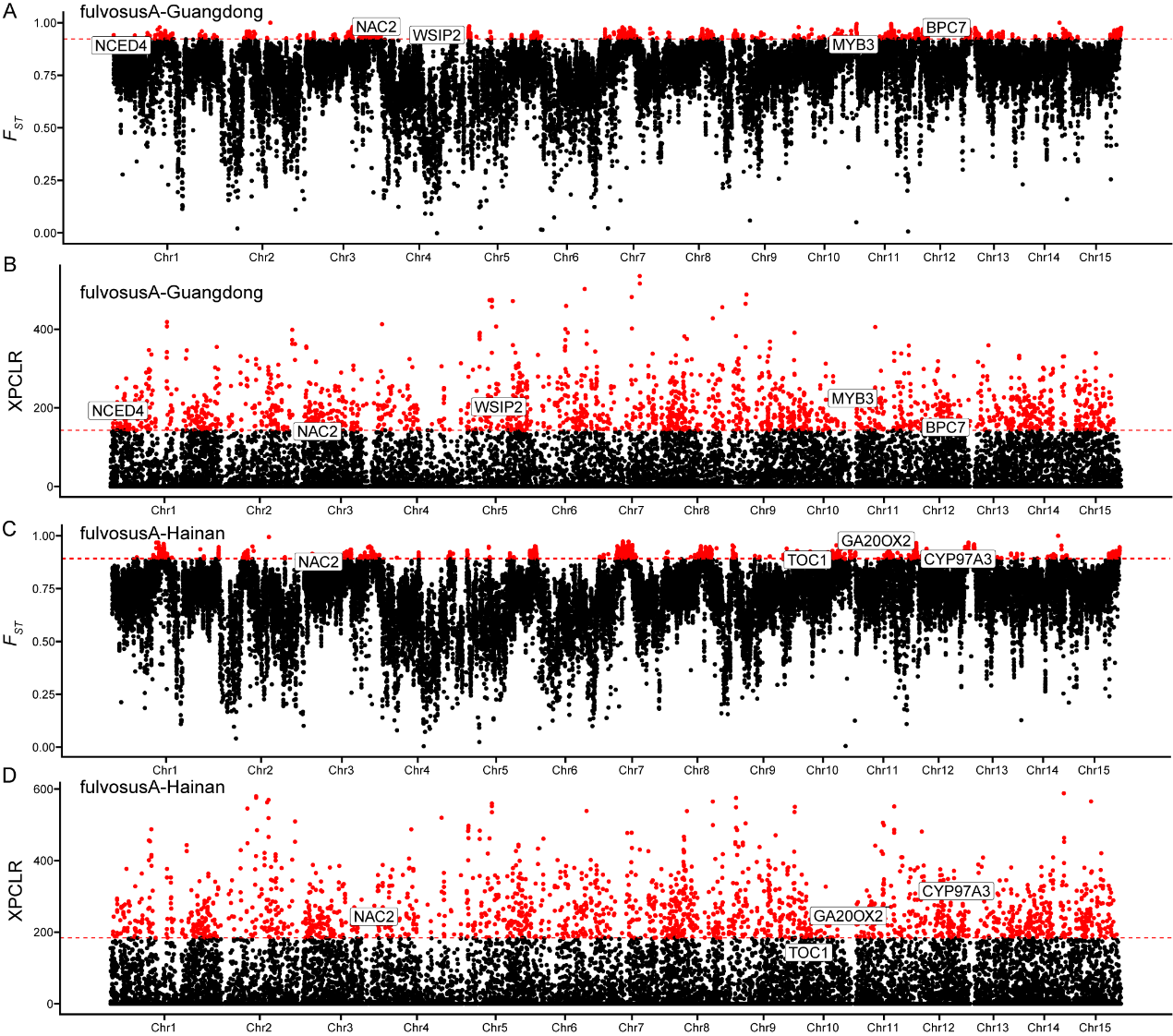
**

**Supplementary Fig. 6 Selective signals between fulvosusA and two litchi subgroups**

**A.** Genome-wide genetic differentiation index between fulvosusA and Guangdong subgroup. **B**. Selective sweeps between fulvosusA and Guangdong subgroup. **C.** Genome-wide genetic differentiation index between fulvosusA and Hainan subgroup. **D**. Selective sweeps between fulvosusA and Hainan subgroup.

**
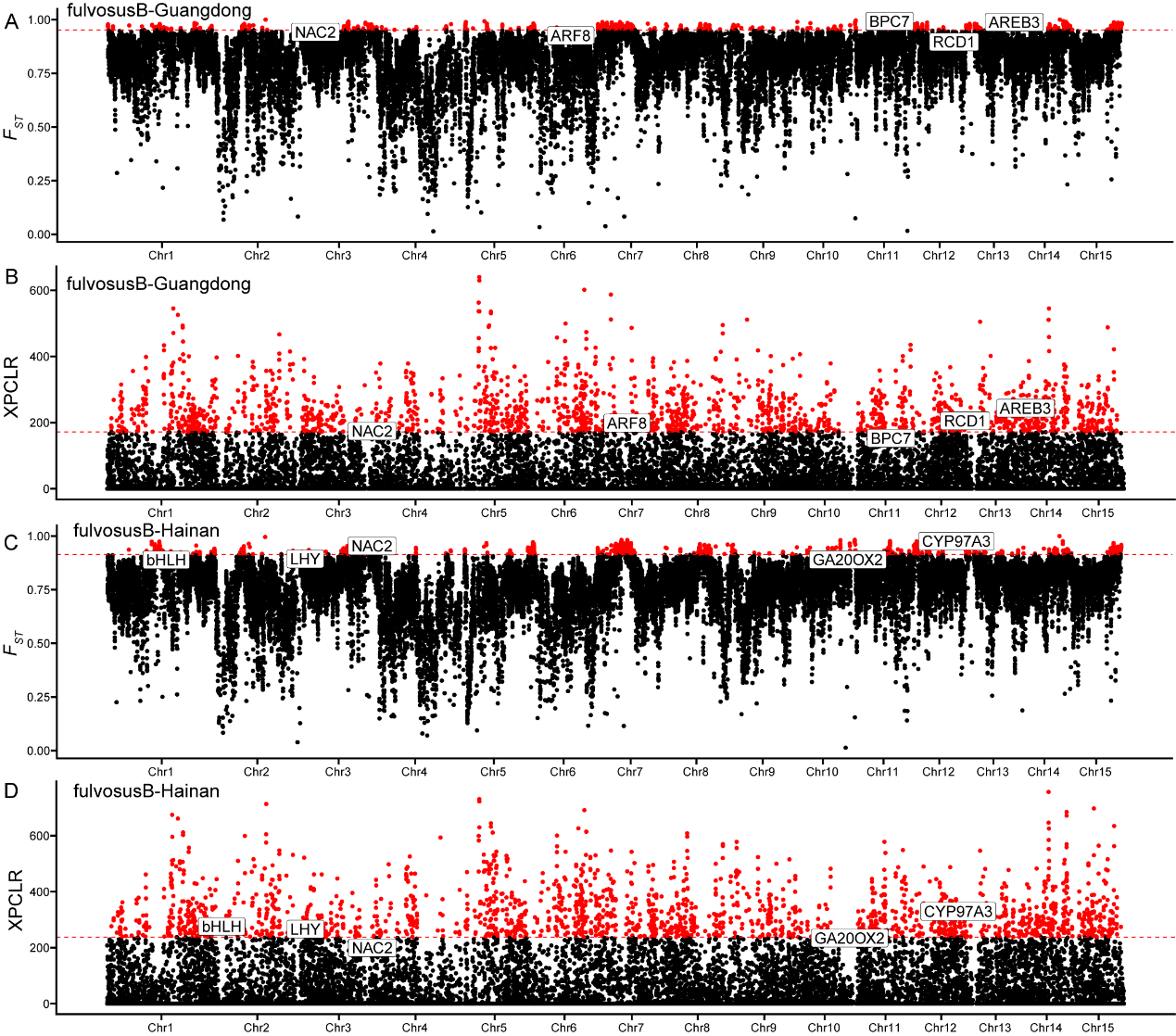
**

**Supplementary Fig. 7 Selective signals between fulvosusB and two litchi subgroups**

**A.** Genome-wide genetic differentiation index between fulvosusB and Guangdong subgroup. **B**. Selective sweeps between fulvosusB and Guangdong subgroup. **C.** Genome-wide genetic differentiation index between fulvosusB and Hainan subgroup. **D**. Selective sweeps between fulvosusB and Hainan subgroup.

**
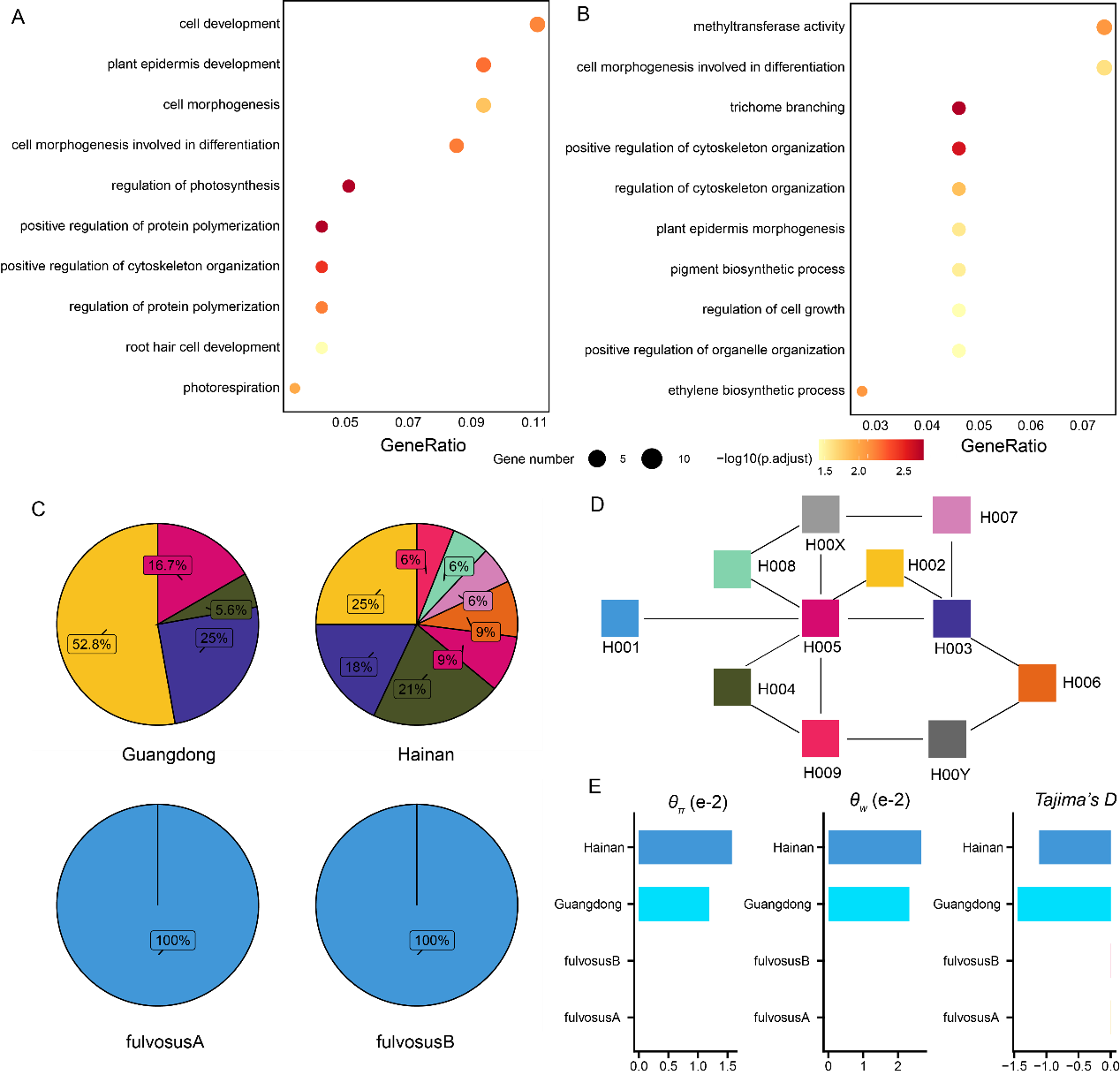
**

**Supplementary Fig. 8 Selective signals between *L. chinensis* var. *fulvosus* and litchi subgroups**

**A**. Gene Ontology enrichment analysis of selective signal genes among fulvosusA, Guangdong and Hainan. **B**. Gene Ontology enrichment analysis of selective signal genes among fulvosusB, Guangdong and Hainan. **C**. Distribution of *NAC2* haplotype in different subgroups. **D**. Haplotype evolution path of *NAC2*. **E**. Genetic parameters of the *NAC2* region in different subgroups.

**
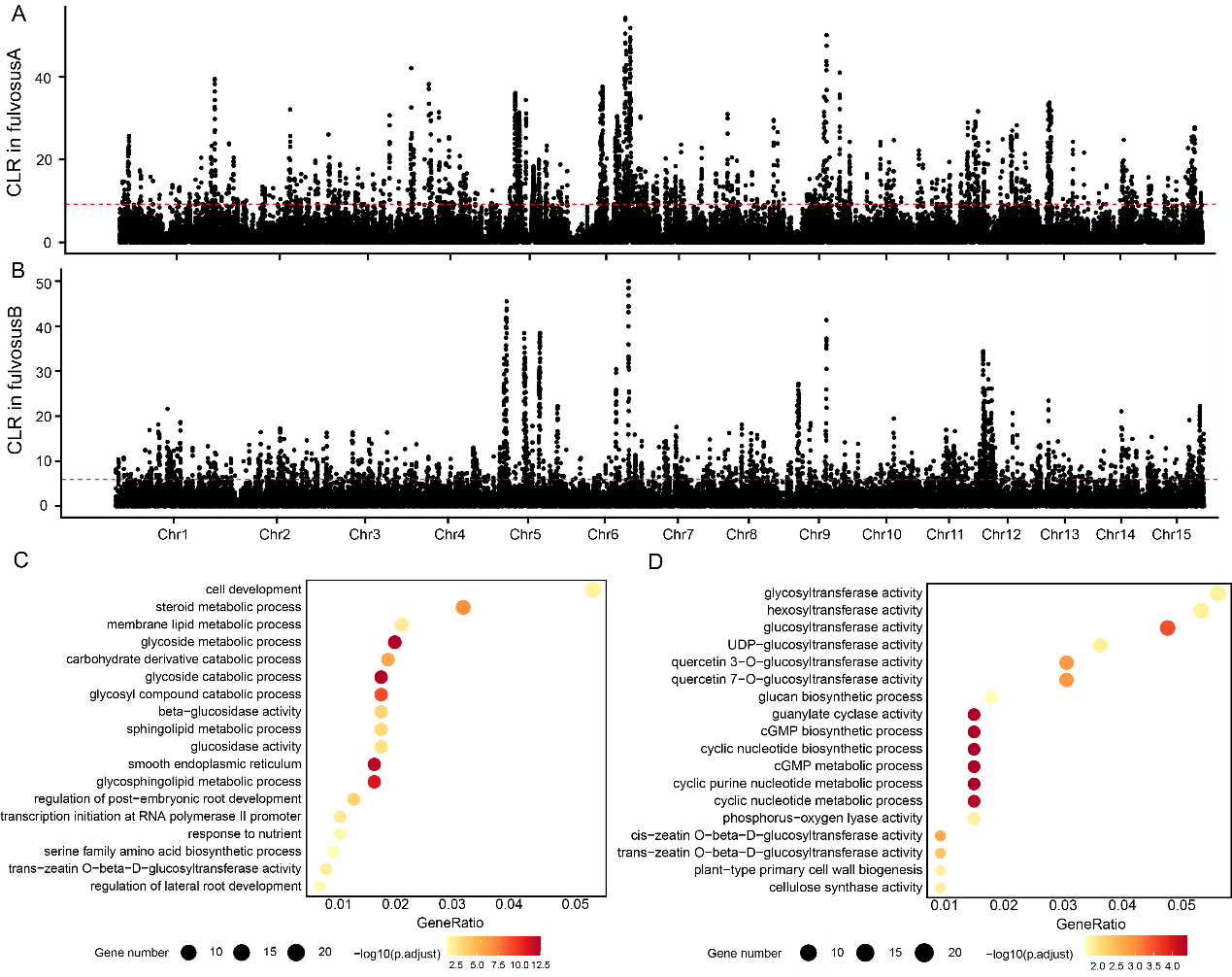
**

**Supplementary Fig. 9 Selective signals in fulvosusA and fulvosusB**

**A.** Selective sweeps in fulvosusA. **B**. Selective sweeps in fulvosusB. **C.** Gene Ontology enrichment analysis of selective genes identified in fulvosusA. **D**. Gene Ontology enrichment analysis of selective genes identified in fulvosusB.

**
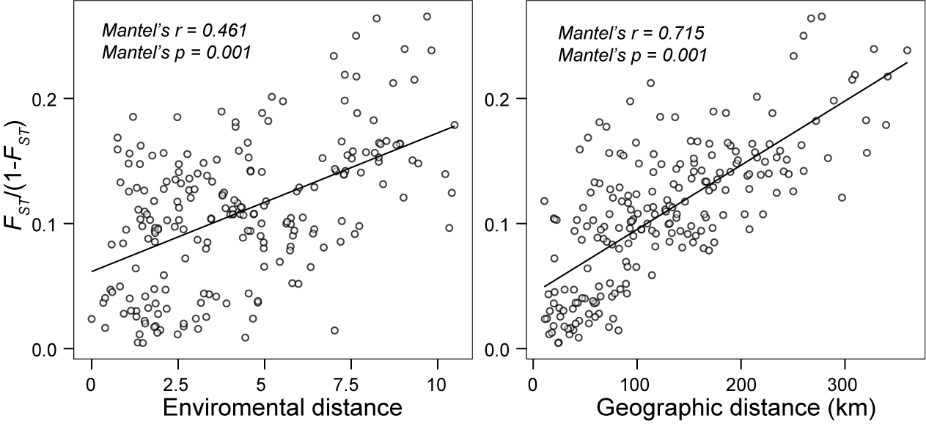
**

**Supplementary Fig. 10 Isolation-by-environment and isolation-by-distance analysis for *L. chinensis* var. *fulvosus***

**
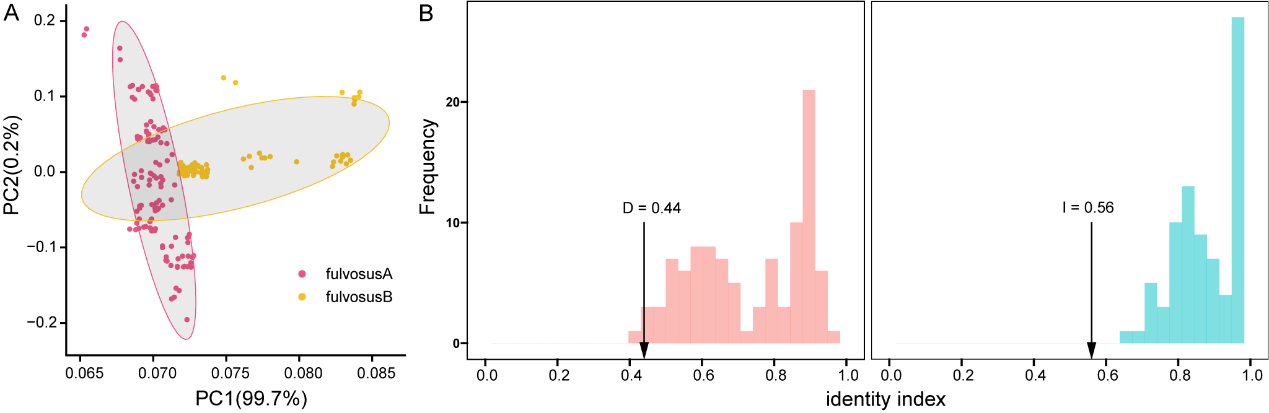
**

**Supplementary Fig. 11 Ecological differentiation among the two subgroups in *L. chinensis* var. *fulvosus***

A. PCA of 19 bioclimatic variables. **B.** The niche identity test for each pairwise comparison measured using Schoener's D and Hellinger distance I in ENMTools.


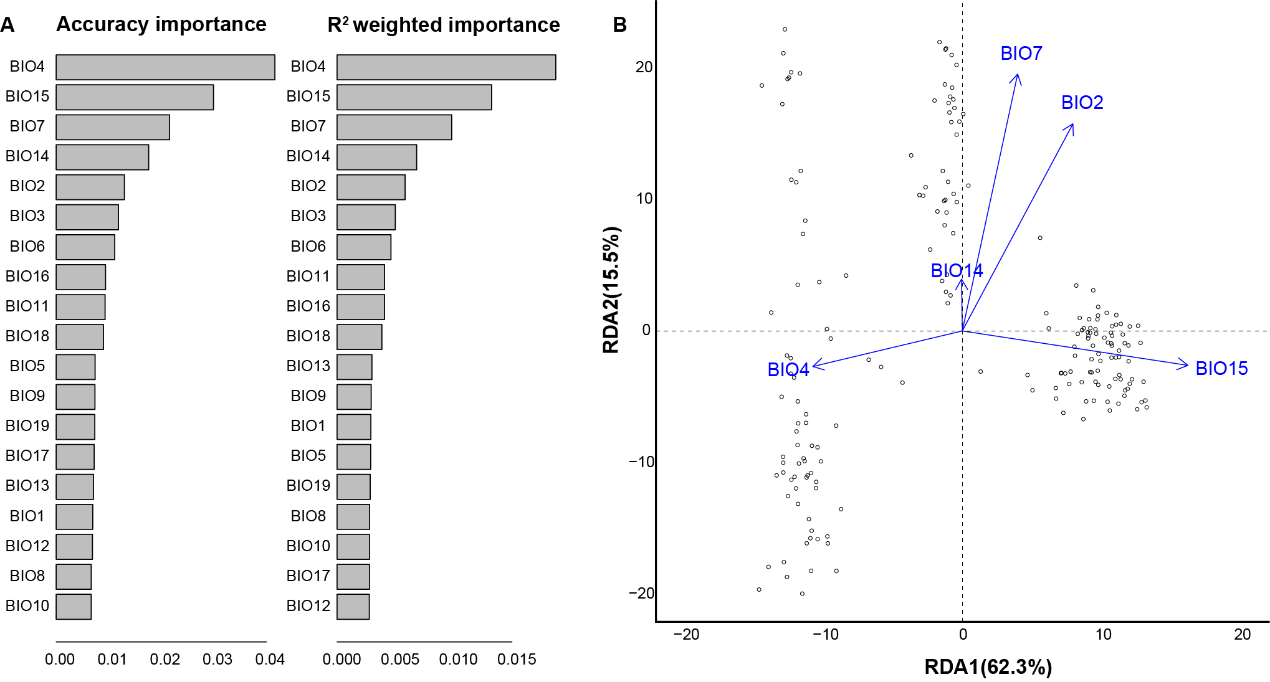


**Supplementary Fig. 12 Construction of random forest model for local environmental adaptability**

**A**. Importance of environmental variables in random forest models of local environmental adaptability. **B**. RDA analysis of the five most important environmental variables.


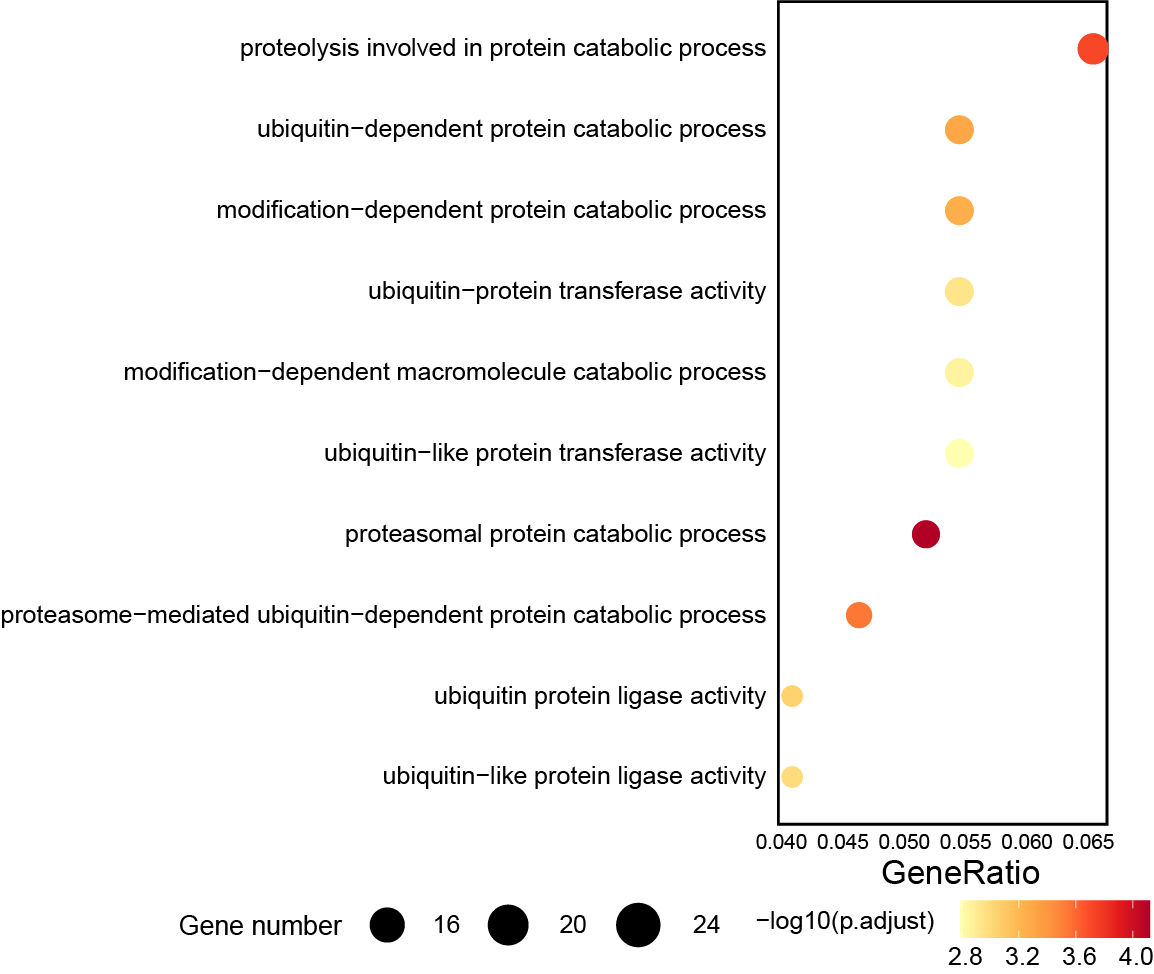


**Supplementary Fig. 13** **Gene Ontology enrichment analysis of core adaptive genes identified by LFMM and RDA.**


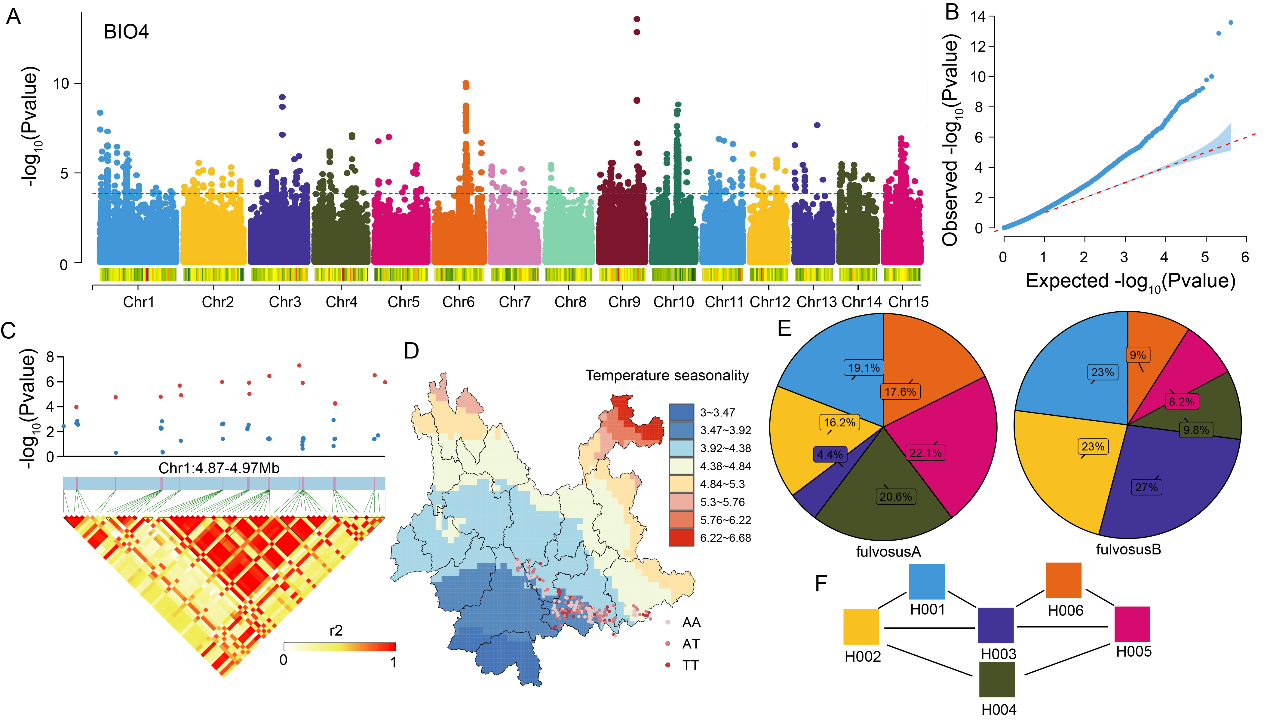


**Supplementary Fig. 14 Genome-wide screening of the loci associated with** **temperature seasonality**

**A**. Manhattan plot for variants with temperature seasonality (BIO4). Red dashed line represents the FDR correction, adjusted *P = 0.05*. **B**. QQ plot for variants with temperature seasonality (BIO4). **C**. Local manhattan plot of candidate adaptive SNP nearby LITCHI014753 associated with BIO4. **D**. Allele distribution of candidate adaptive SNP associated with BIO4. **E**. Distribution of LITCHI014753 haplotype in two subgroups. **F**. Haplotype evolution path of LITCHI014753.


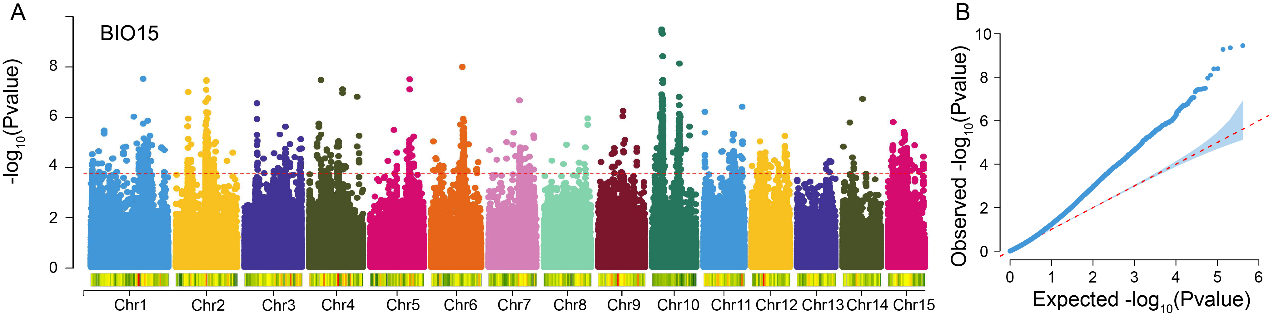


**Supplementary Fig. 15 Genome-wide screening of the loci associated with precipitation seasonality**

**A**. Manhattan plot for variants with precipitation seasonality (BIO15). Red dashed line represents the FDR correction, adjusted *P = 0.05*. **B**. QQ plot for variants with precipitation seasonality (BIO15).


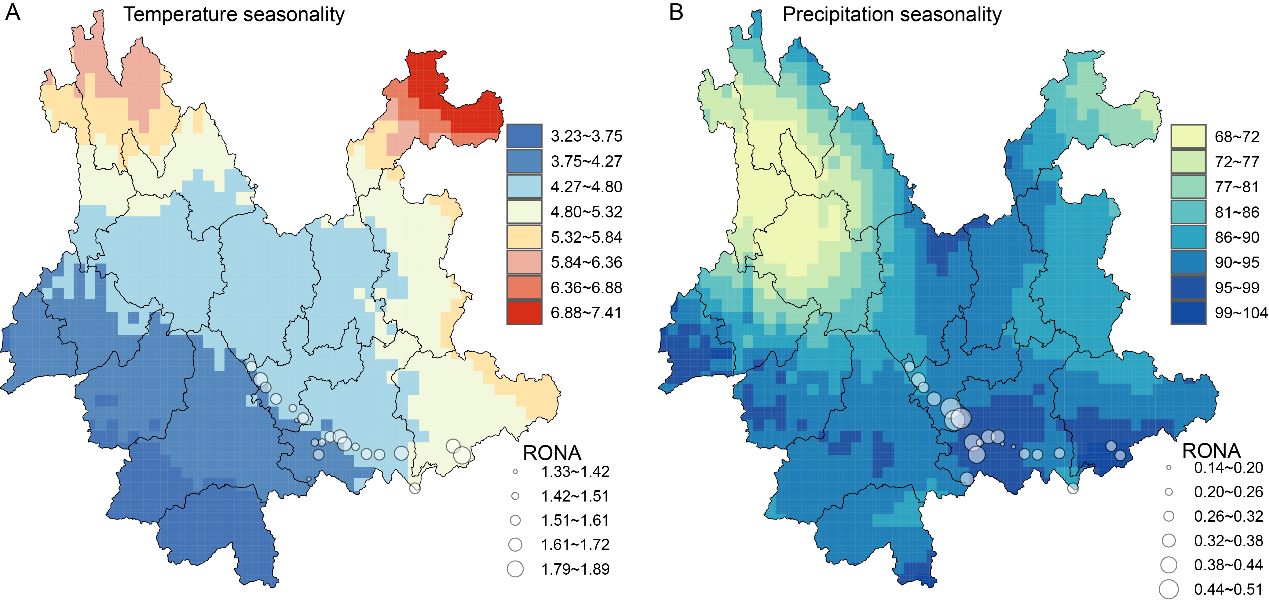


**Supplementary Fig. 16 Genetic vulnerability to future climate change under SSP245 and SSP585**

**A**. The RONA estimates of BIO4 for the 21 groups under the SSP585 climate scenarios in 2081-2100. **B**. The RONA estimates of BIO15 for the 21 groups under the SSP585 climate scenarios in 2081-2100.


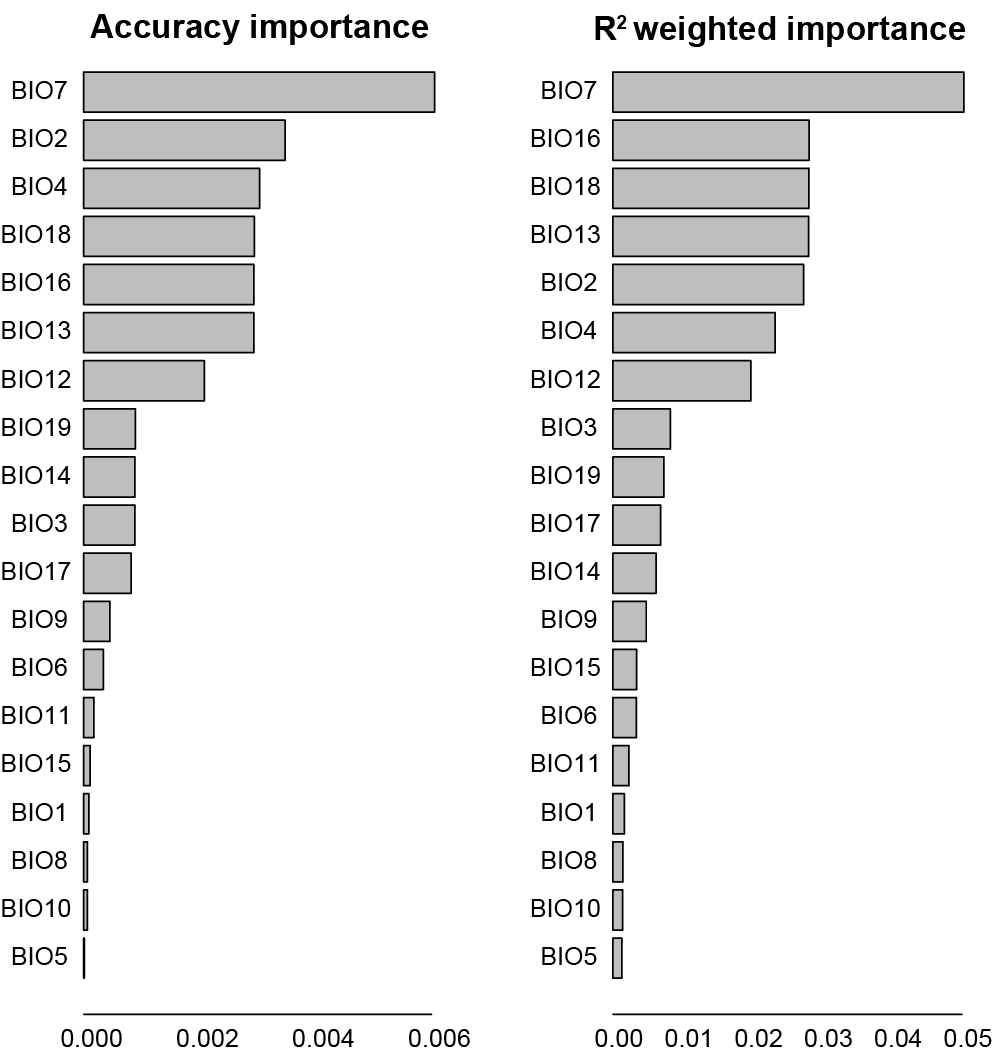


**Supplementary Fig. 17 Importance of environmental variables in random forest models of genetic vulnerability**


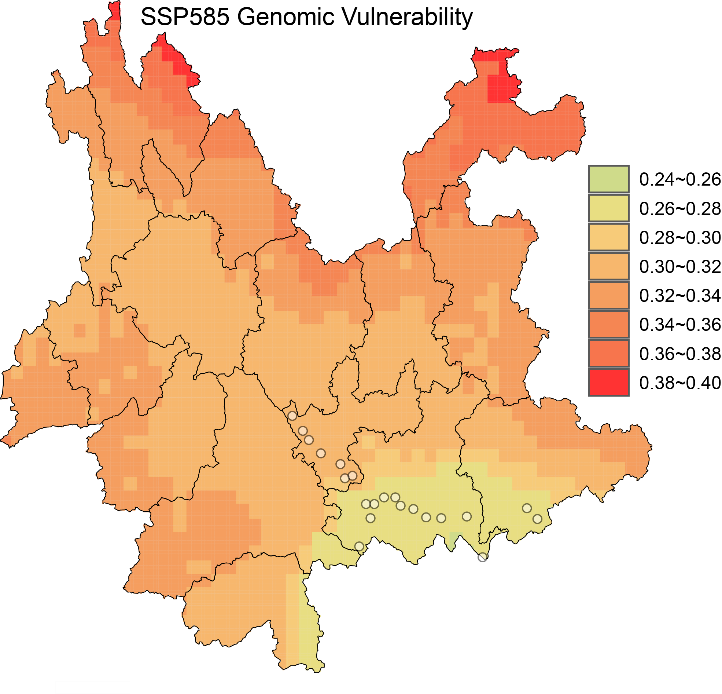


**Supplementary Fig. 18 The genetic offset under the SSP585 climate scenarios in 2081-2100**


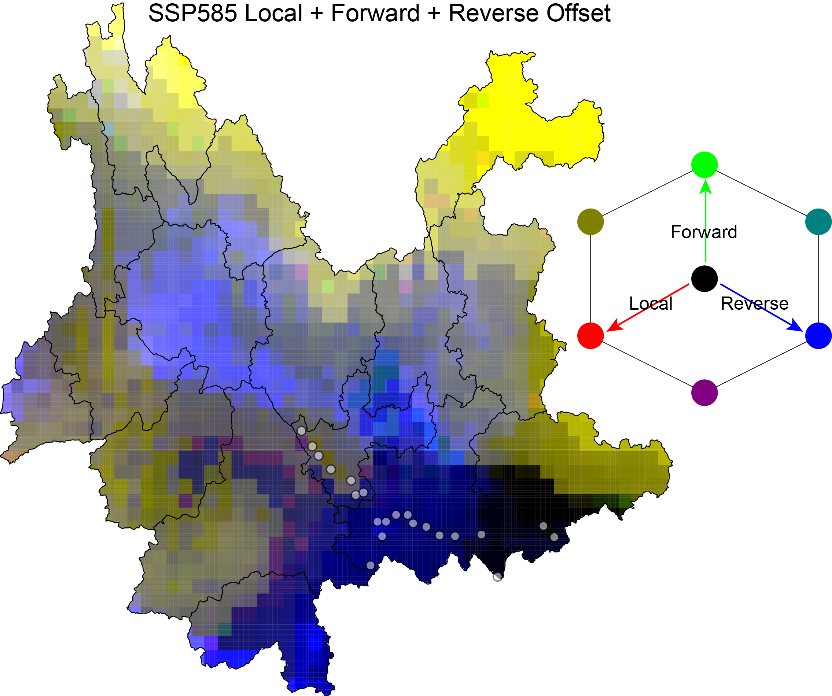


**Supplementary Fig. 19 The RGB map of local (red), forward (green), and reverse (blue) offset under the SSP585 climate scenarios in 2081-2100**
